# Multiple single cannulation technique of arteriovenous fistula: A randomized controlled trial

**DOI:** 10.1111/hdi.12962

**Published:** 2021-07-06

**Authors:** Ricardo Peralta, João Fazendeiro Matos, Bruno Pinto, Pedro Gonçalves, Rui Sousa, Carla Felix, Helena Carvalho, José Vinhas, Pedro Ponce

**Affiliations:** ^1^ NephroCare Portugal Fresenius Medical Care Portugal Maia Porto Portugal; ^2^ NephroCare Viseu Fresenius Medical Care Viseu Viseu Portugal; ^3^ Instituto Universitário de Lisboa (ISCTE‐IUL), Centro de Investigação e Estudos de Sociologia (CIES‐IUL) Lisbon Portugal; ^4^ Nephrology Department Setúbal Hospital Centre Setúbal Portugal; ^5^ NephroCare Portugal Fresenius Medical Care Portugal Lisbon Portugal

**Keywords:** arteriovenous fistula, buttonhole technique, cannulation, hemodialysis, rope‐ladder technique

## Abstract

**Introduction:**

Despite the impact needling has had on vascular access survival and patient outcome, there is no universal or standardized method proposed for proper cannulation. Rigorous studies are needed, examining cannulation practices, and challenges to achieving complication‐free cannulation.

**Methods:**

This randomized, open‐label trial was conducted at 18 dialysis units owned by a large private dialysis provider operating in Portugal. Eligible patients were adults on chronic hemodialysis, with a new arteriovenous fistula (AVF); cannulated for at least 4 weeks complication‐free. Patients were randomly assigned in a 1:1 ratio to one of three cannulation techniques (CT): Multiple Single cannulation Technique (MuST), rope‐ladder (RLC), and buttonhole (BHC). The primary endpoint was AVF primary patency at 1 year.

**Findings:**

One hundred seventy‐two patients were enrolled between March 2014 and March 2017. Fifty‐nine patients were allocated to MuST, 56 to RLC, and 57 to BHC.

MuST and RLC were associated with a better AVF primary patency than BHC. Primary patency at 12 months was 76.3% in MuST, 59.6% in BHC, and 76.8% in RLC group. Mean AVF survival times were 10.5 months (95% CI = 9.6, 11.3) in the MuST group, 10.4 months (95% CI = 9.5, 11.2) in RLC, and 9.5 months (95% CI = 8.6, 10.4) in BHC. BHC was a significant risk predictor for AVF survival with 2.13 times more events than the other two CT (HR 2.13; 95% CI = 1.07, 4.21; *p* = 0.03).

**Discussion:**

MuST was easy to implement without a diagram and there is no need to use blunt needles. This study showed MuST was efficacious and safe in maintaining the longevity of AVF in dialysis patients.

## INTRODUCTION

Vascular access (VA) is an essential component of hemodialysis (HD) for end‐stage kidney disease (ESKD) patients, relying on sustained extracorporeal circulation.^1^ The ideal VA should allow cannulation using two needles, deliver a minimum blood flow of at least 300 ml/min through the artificial kidney, be resistant to infection and thrombosis and have minimum adverse events.^2^ However, the preservation of a VA free from complications remains one of the most challenging aspects of renal replacement therapy.

The morbidity and mortality of patients in HD are directly related to the type of VA. The risk of infectious complications at the start of HD is four times greater when using a central venous catheter (CVC) than an arteriovenous fistula (AVF).^3^

An autogenous AVF is therefore the preferred type of dialysis access. However, maintenance of the VA depends not only on the blood vessels' quality and the used surgical technique, but also on the way in which the VA is cannulated.^1^ AVF cannulation methods are still considered to be an art; a procedure that reflects local unit practices and nursing skills.^4^ Interestingly, despite the impact needling has had on VA survival and patient outcome,^4^ there is no universal or standardized method proposed for proper cannulation.^2^

However, the current situation in the real world is disappointing. The most‐used cannulation technique (CT) was the area method (65.8%) in 171 HD units in 9 countries of the European Union.^4^ The true rate may be underestimated and may extend to 100% of patients.^5^ Although this CT is not recommended, it is predominantly used in daily practice, even when there is a prescribed protocol to use rope‐ladder cannulation (RLC).^6^

Several randomized controlled trials (RCTs) have compared the complication rate and AVF primary patency between buttonhole cannulation (BHC) and RLC. Each study used a different definition of RLC as the: usual practice of RL rotation technique,^7^ standard needling,^8^, ^9^ and conventional different‐site technique,^10^ seeming to presume this is area CT or different puncture methods. Concomitantly, they defined BHC in detail but omitted to describe how they used CT in the control group.

The relative benefits and risks of BHC versus RLC are unknown and have not yet been explored in a RCT in patients with new AVF. The need for more rigorous studies in this area, examining cannulation practices and achieving complication‐free cannulation, is mentioned in the recently published *Kidney Disease Outcomes Quality Initiative* (KDOQI).^11^

Interestingly, a new approach to AVF cannulation with noticeable benefits for patients has been used since 2013, the Multiple Single cannulation Technique (MuST).^12^ This CT consists of rotating the same six specific cannulation sites, three arterial and three venous, through the three treatment days of each week.

The purpose of this study was to determine the efficacy of MuST compared to RLC and BHC.

## MATERIALS AND METHODS

### Study design

This randomized open‐label trial was conducted at 18 dialysis units belonging to a large private dialysis provider operating in Portugal.

### Patients

Eligible patients were adults on ESKD, with AVF for VA, dialyzed three times per week with a 4‐h schedule, undergoing online hemodiafiltration.

As inclusion criteria, all enrolled patients were >18 years old with a new (unused) AVF; cannulated for at least 4 weeks with no complications, having at time of randomization an access blood flow (Qa) ≥500 ml/min, with tracks that allowed cannulations over the length of the vein with at least 6 cm of distance between needle bevels, or two distinct areas of 3 cm in length. During the study, 15G dialysis needles were used as standard, whereas for BHC blunt needles were used. Patients using anesthetic creams at cannulation sites were excluded.

### Procedures

The study consisted of two phases. The first phase comprised comprehensive training of all the clinics' nursing teams in the MuST method before patient selection. In the second phase, patients were selected according to the inclusion criteria and were only randomized after AVF maturation and a screening phase of 4 weeks for vessel stabilization. Patients were centrally randomized to either MuST, RLC, or BHC via a computer‐generated model using randomly selected patient blocks of equal sizes. It was not possible to blind patients and nurses due to the intervention characteristics; therefore, only the evaluation investigator was blinded.

**FIGURE 1:**
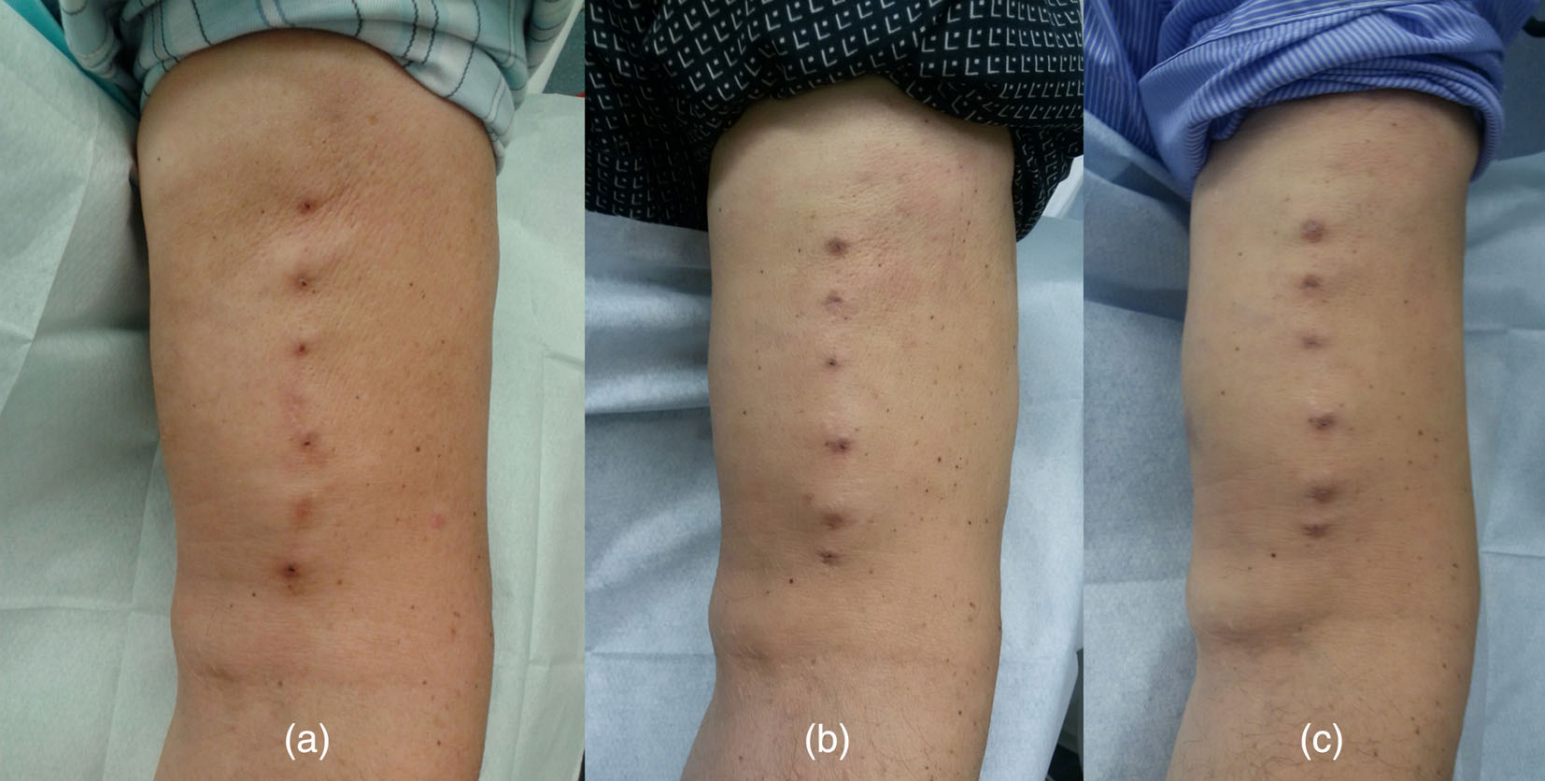
Brachio‐cephalic arteriovenous fistula at left upper arm. Established Multiple Single cannulation Technique (MuST) in a patient with new fistula. (a) MuST at the beginning of the cannulation after 4 weeks. (b) After 18 months of use. (c) Arteriovenous fistula cannulation sites with MuST use after 30 months [Color figure can be viewed at wileyonlinelibrary.com]

**FIGURE 2:**
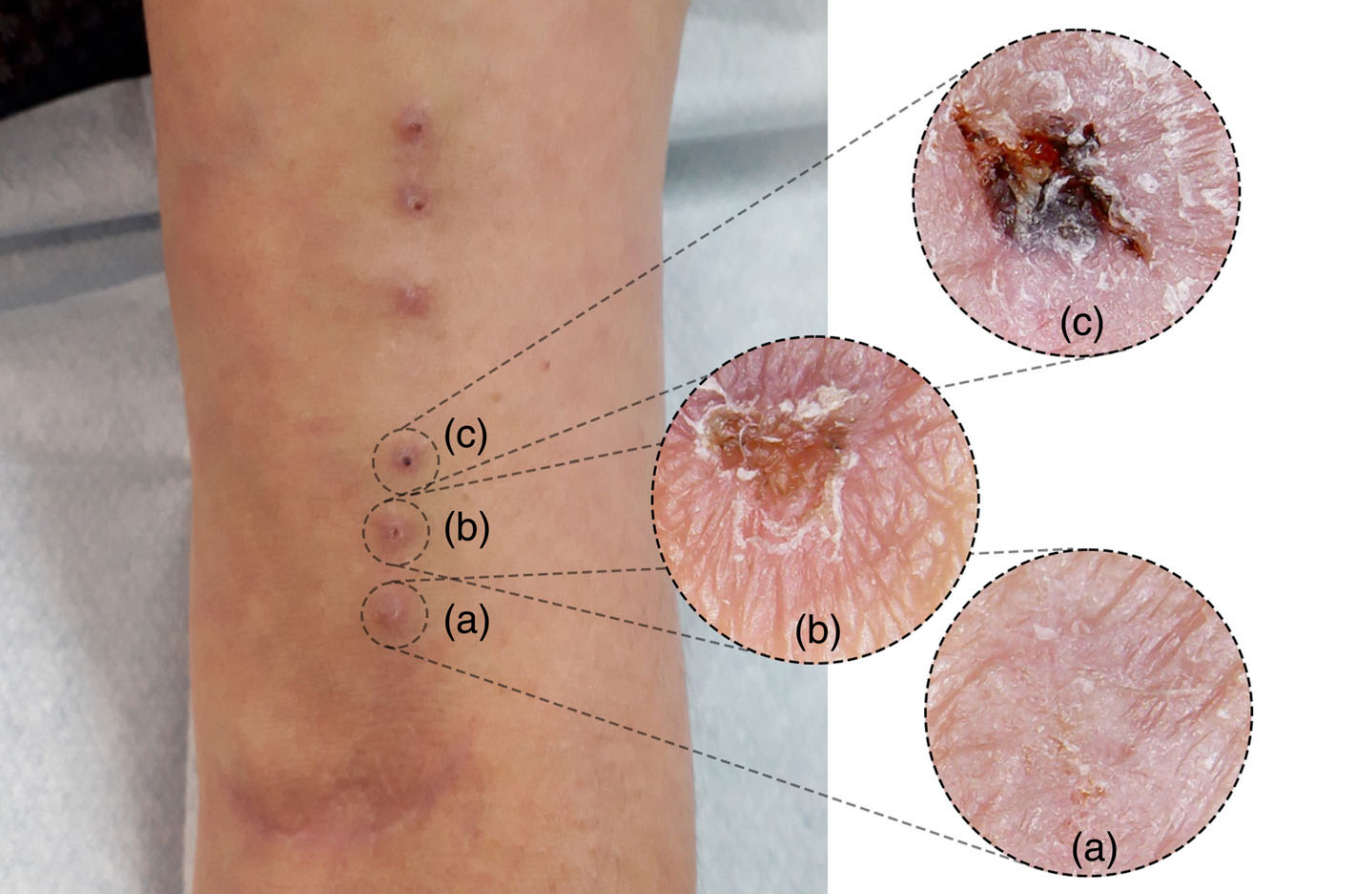
Multiple Single cannulation Technique (MuST) with six sites, three arterial and three venous. Healing of different cannulation sites in MuST. (a) first day of the week, (b) second day of the week, and (c) third day of the week [Color figure can be viewed at wileyonlinelibrary.com]

For MuST and RLC, individual diagrams of the AVF were created and attached to the patient's file for easy consultation. MuST incorporates both RLC and BHC methods by using the entire length of the VA available through progressive rotation, and by having three specific cannulation sites for each day of the week, meaning that each site is only cannulated once a week (Figure 1). This hybrid method allows the cannulation site to heal between cannulations (Figure 2). The selected cannulation sites were marked with a dermographic pen during the first two weeks. Two areas of arterial and venous cannulation were created, with three cannulation points each at least 1 cm apart. For MuST CT, standard dialysis needles were used.

The RLC was implemented according to the diagram created. In the BHC group, disinfection was performed before and after removing the scabs at the cannulation sites. Subcutaneous' tunnel construction was carried out by experienced nurses to maintain its integrity. Before entering the treatment room, patients washed the skin area above the AVF. A sterile cannulation set was used with all the disposables needed for the procedure. Each nurse had a trolley to support connection and disconnection the patient and an automatic dispenser for hand hygiene.

Before cannulation, physical examination of the AVF was performed at every dialysis session by the supervising nurse and all parameters were recorded in a specific VA database. Whenever a change in the physical or dynamic examination was identified, the head nurse and respective nephrologist were informed. All interventions required for the AVF were scheduled with the VA center. The referral causes, and procedures were recorded in the same database. To complete the information, a report with several treatment parameters was created for each patient during the follow‐up period.

Qa was regularly measured by thermodilution via the BTM® (Blood Temperature Monitor) from 5008 dialysis machines (Fresenius Medical Care [FMC], Bad Homburg, Germany), using the model conceived by Krivitski,^13^, ^14^ according to an already pre‐established VA' surveillance and monitoring program in our network.

Chlorhexidine‐based antiseptic solution or 70% alcohol solution was used to disinfect the skin at the cannulation sites. Photographic recording was performed every 3 months to assess the widening of the vessel.

### Outcomes

The primary endpoint was AVF primary patency at 1 year, determined by the percentage of fistulas in use from the beginning of the study to the date of the first clinical intervention for angioplasty or vascular surgery (unassisted patency),^15^, ^16^ or abandonment of the CT due to difficulties in execution or patient's refusal due to pain.

A fistula that was used unsuccessfully, with patency or not, was considered a dysfunctional AVF.^15^ The day that access was permanently unusable for cannulation was considered AVF abandonment.^1^

Referral for endovascular intervention was based on one or more indicators of AVF dysfunction^15^, ^16^:

- Changes in physical examination (changes in thrill, abnormal development of aneurysm or progressive swelling of the AV limb);
- Qa <400 ml/min (according to the established VA monitoring program);
- Increased hemostasis time (>10 min);
- Cannulation failure: failure or inability to insert the dialysis needles^1^;
- Decreased dialysis efficacy: deficient dose of HD, spKt/V <1.2 or substitution fluid <21 L.^1^ The dialysis dose (spKt/V) was calculated based on the ion clearance obtained by the module Online Clearance Monitor (OCM®, FMC) of the dialysis 5008 CorDiax machine and the volume of urea distribution “V” derives from the determination by bioimpedance through the Body Composition Monitor—FMC.

Referral for surgical intervention was based on one or more indicators of AVF dysfunction^15^:

- Rupture of the AVF wall or thrombosis;
- Progressive development of aneurysm;
- Acute bleeding;
- Local AVF infection;
- Deficient distal perfusion with signs of ischaemia.

The secondary outcome was the proportion of AVF complications, including inflammatory signs at the AVF cannulation sites (defined by the presence of one or more signs: flushing, oedema, or local exudate)^16^; local AVF infection (defined by the presence of exudate at the cannulation site with positive bacteriological culture and AVF‐related bacteremia, confirmed with a positive result by blood culture); and aneurysms' development (as determined by the presence of an enlarged segment of the arterialized vein three times the diameter of the segment considered normal, which means a segment with a width ≥1.8 mm).^17^

All data were recorded in each patient's file in the EuCliD database. Other indicators of the physical examination and difficulties in performing the CT were recorded on the specific worksheet.

### Statistical analysis

Primarily, a descriptive analysis was conducted. Continuous variables were reported with mean and interquartile range (IQR). For categorical variables, the frequency with the percentage is presented. Comparisons of baseline characteristics were carried out using one‐way ANOVA, analysis of homogeneity between groups or chi‐squared tests for categorial variables. Because the percentage of missing cases was residual, the procedure was to exclude cases listwise. The analysis focused on the comparison of the frequency of events reported for angiography and vascular surgery between MuST and the remaining two CT.

Intention‐to‐treat survival analysis was used to compare time to AVF functional patency (primary outcome) between the three cannulation groups using Kaplan–Meier formula and survival curves plotted with log‐rank test as the primary comparison. Participants were censored when referred to another clinic, hospitalized, modified treatment modality, transplant, death, or completion of the study (right censoring). A Cox proportional hazards model was also used to estimate hazard ratio (HR) with 95% confidence intervals (CIs). To control the type I error at 5%, a hierarchical multiple testing procedure was used across the primary and secondary analyses. Tests were made on the following order: primary patency—inflammatory signs at the AVF cannulation site—local AVF infection—development of aneurysms.

Efficacy data were analyzed in the modified intention‐to‐treat population, which including all randomized patients.

Results were considered significant when *p* <0.05. All the statistical analysis was performed using SPSS (version 23; IBM, Armonk, NY).

### Compliance with ethical standards

This study was approved by the institution's ethical committee in accordance to national and the Declaration of Helsinki requirements. All patients signed an informed consent and were free to withdraw from the study at any time.

## RESULTS

### Patients' characteristics and treatment data

Patient recruitment occurred between March 2014 and March 2017, follow‐up and data collection continued until March 2018.

Baseline demographics were well balanced between study groups. For this study 172 subjects were enrolled, 59 in MuST, 56 in RLC, and 57 in BHC. Mean age was 67.73 (SD = 14.24) years old (range from 31 to 91), mostly males (n = 134, 77.9%). Table 1 summarizes patients' demographic characteristics, ESKD etiology, comorbidities, laboratory parameters, and previous VA in the three CT. Mean AVF vintage was 9.80 (2.02–9.50) months after its construction, with 50% having less than 3 months of maturation. It is important to note that this was the first internal VA for 132 (76.7%) of patients. A CVC was implanted in 93 (54.1%) patients before the construction of the active AVF. Mean dialysis vintage time of the participants before entering the study was 17.15 (2–8) months, median of 4 months. Diabetic nephropathy and arterial hypertension were the two main causes of ESKD; 58 (33.7%) and 25 (14.5%), respectively. The most frequent comorbidities were heart disease and hypertension (61.6%), diabetes mellitus (41.9%), and peripheral circulatory disease (30.8%). However, there were differences between groups in disease characteristics but without statistical significance. For example, a longer dialysis vintage (22.8 months vs. 15.3 months in BHC, and 13.0 months in RLC) and a greater proportion of diabetes (49.2 vs. 38.6% in BHC, and 37.5% in RLC), was observed in the MuST group.

**TABLE 1:** Patient baseline characteristics according to cannulation technique

Variables	MuST (n = 59)	Buttonhole (n = 57)	Rope‐ladder (n = 56)	*p*
*Demographics*				
Age (years), mean (IQR)	68.46 (57–80)	68.74 (56–79.50)	65.95 (58.25–79.75)	0.521^a^
Male, *n* (%)	47 (35.1)	42 (31.3)	45 (33.6)	0.640^b^
Dry weight (kg), mean (IQR)	73.33 (62–82)	71.54 (62.50–79.25)	71.32 (63–81.87)	0.750^a^
AVF vintage (months), mean (IQR)	8.79 (1.9–6.30)	11.27 (2.10–8.30)	9.37 (2–10.52)	0.709^a^
Dialysis vintage, (months), mean (IQR)	22.84 (2–10)	15.31 (2–8)	13.01 (1–7)	0.432^a^
Qa_BTM (ml/min)	1267 (810–1750)	1287 (820–1920)	1257 (830–1700)	0.949^a^
Anticoagulant (UI/Kg)	55.14 (52.94–65.57)	58.30 (46.48–69.08)	57.94 (46.48–67.37)	0.628^a^
*Cause of ESKD n (%)*				
Diabetes	21 (35)	21 (36.8)	16 (28.6)	
Hypertension/vascular	7 (11.9)	8 (14)	10 (17.9)	
Polycystic kidney disease	6 (10.2)	5 (8.8)	5 (8.9)	
Glomerulonephritis	3 (5.1)	6 (10.5)	5 (8.9)	
Hypoplasia/dysplasia	0	3 (5.3)	1 (1.8)	
Cause unknown	11 (18.6)	8 (14)	11 (19.6)	
Other known cause	11 (18.6)	6 (10.5)	8 (14.3)	
*Comorbidities n (%)*				
Diabetes	29 (49.2)	22 (38.6)	21 (37.5)	0.372^b^
Heart disease	34 (57.6)	36 (63.2)	36 (64.3)	0.732^b^
Peripheral vascular disease	17 (28.8)	21 (36.8)	15 (26.8)	0.470^b^
Pulmonary disease	7 (11.9)	7 (12.3)	9 (16.1)	0.768^b^
Digestive tract disease	6 (10.2)	7 (12.3)	8 (14.3)	0.797^b^
Endocrine/nutritional diseases	6 (10.6)	11 (19.3)	8 (14.3)	0.377^b^
Cancer	9 (15.3)	7 (12.3)	10 (17.9)	0.710^b^
Other	11 (18.6)	9 (15.9)	3 (5.4)	0.090^b^
*Laboratory values*				
Hematocrit (%), (IQR)	32.99 (31.2–35.30)	34.01 (32–35.55)	34.14 (31.90–36.55)	0.168^a^
Albumin (g/dl), (IQR)	3.85 (3.60–4.10)	3.89 (3.70–4.20)	3.89 (3.65–4.10)	0.842^a^
*Previous vascular accesses n (%)*				
Previously constructed vascular access (yes)	15 (37.5)	14 (35)	11 (27.5)	0.734^b^
CVC previously implanted (yes)	36 (38.7)	29 (31.2)	28 (30.1)	0.416^b^

*Note*: % presented are related to the frequencies evaluated within the respective class of cannulation techniques. For continuous variables, means and the interquartile range (IQR) are shown. For categorical variables, the frequency and percentage are presented.

Abbreviations: BTM, blood temperature monitor; CVC, central venous catheter; Qa BTM, blood flow measured by BTM.

^a^ One‐way ANOVA to compare the three groups with continuous outcomes.

^b^ *Χ* ^2^ test for categorical variables.

**FIGURE 3:**
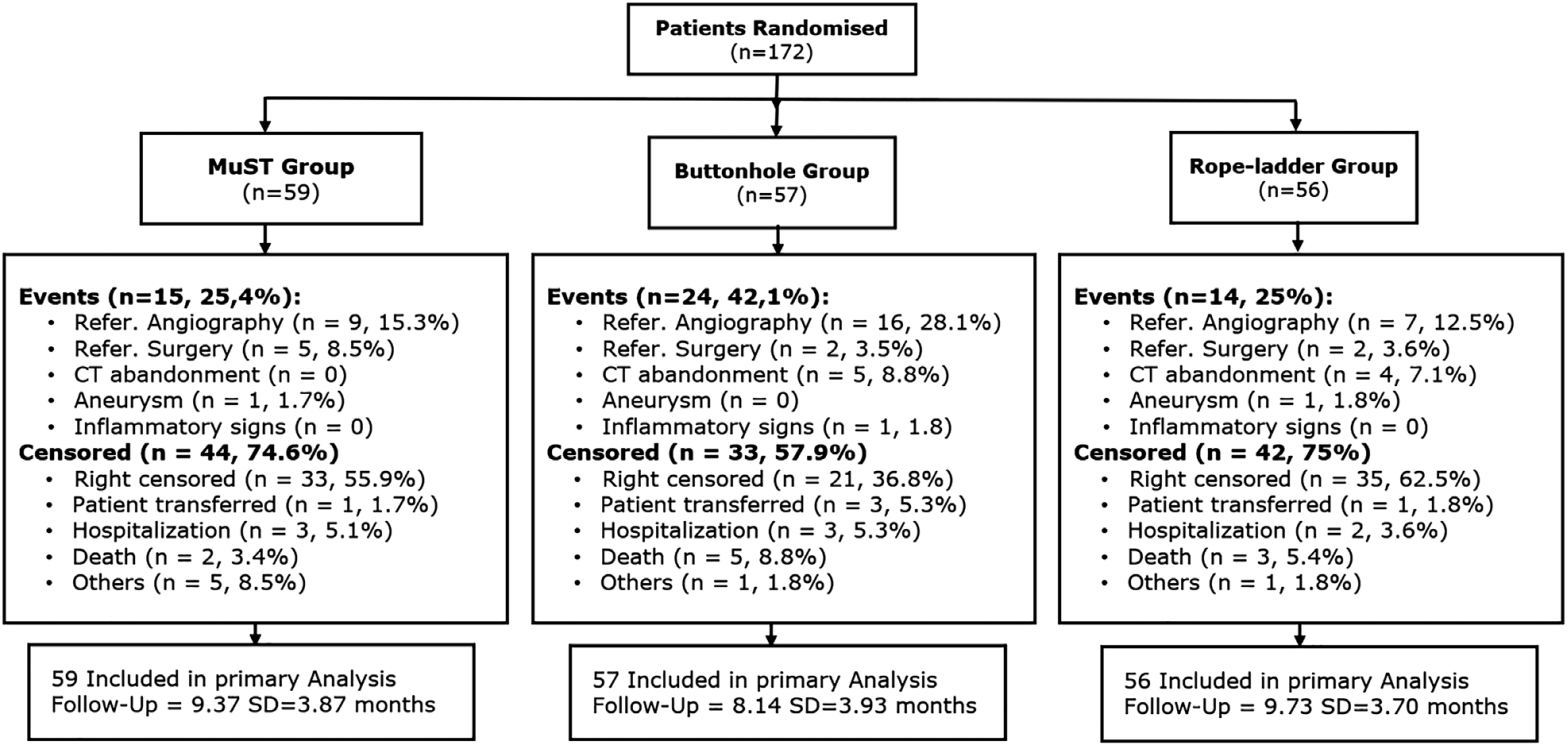
Patient flow diagram describing randomization and follow‐up. *Note*: the frequencies and % refer to the totals within each cannulation technique (CT)

Patients were followed on average 9.08 (SD = 3.87) months, but 89 (51.7%) were right‐censored, with no event occurring. It was observed that MuST and RLC had more patients censored (n = 44, 74.6% and n = 42, 75%) than BHC (n = 33, 57.9%; Figure 3). When comparing the frequency of events between the MuST (n = 15, 25.4%) and the other groups, it was observed that the results were equivalent to the RLC (n = 14, 25%), whereas a higher frequency was reported in the BHC (n = 24, 42.1%).

As observed in Figure 3, BHC is associated with a higher frequency of referral to angiography than the other two CT. The main reference factor was confirmed Qa drop in AVF output in 11 (19.3%) of the patients undergoing this CT. Three participants in the MuST group required surgical revision due to the distal limb ischemia and one patient from each of the remaining CT. Of the 2 AVF complete occlusion access thrombosis, 1 was in the buttonhole and another in the MuST needling group. More referencing factors are provided in Table 2.

**TABLE 2:** Frequency of referencing to angiography and surgery between cannulation techniques

Intervention factors	MuST n = 59 (%)	Buttonhole n = 57 (%)	Rope‐ladder n = 56 (%)
Refer. Angiography (no events)	50 (84.7)	41 (71.9)	49 (87.5)
Qa drop	5 (8.5)	11 (19.3)	2 (3.6)
Changes in physical examination	2 (3.4)	3 (5.3)	2 (3.6)
Decreased dialysis efficacy	2 (3.4)	0	2 (3.6)
Others	0	2 (3.5)	1 (1.8)
Refer. Surgery (no events)	54 (91.5)	55 (96.5)	54 (96.4)
Thrombosis	1 (1.7)	1 (1.8)	0
Development of aneurysm	0	0	1 (1.8)
Deficient distal perfusion	3 (5.1)	1 (1.8)	1 (1.8)
Others	1 (1.7)	0	0

*Note*: the frequencies and % refer to the totals within each cannulation technique (CT).

### Primary outcome

AVF mean survival follow‐up period between the three groups was 10.49 months (95% CI = 9.64, 11.33) in MuST versus BHC 9.47 months (95% CI = 8.55, 10.39) and RLC 10.38 months (95% CI = 9.51, 11.24). Primary patency at 12 months was 76.3%, in the MuST group and 59.6 and 76.8%, respectively, in the BHC and RLC groups (*p* = 0.72).

**FIGURE 4:**
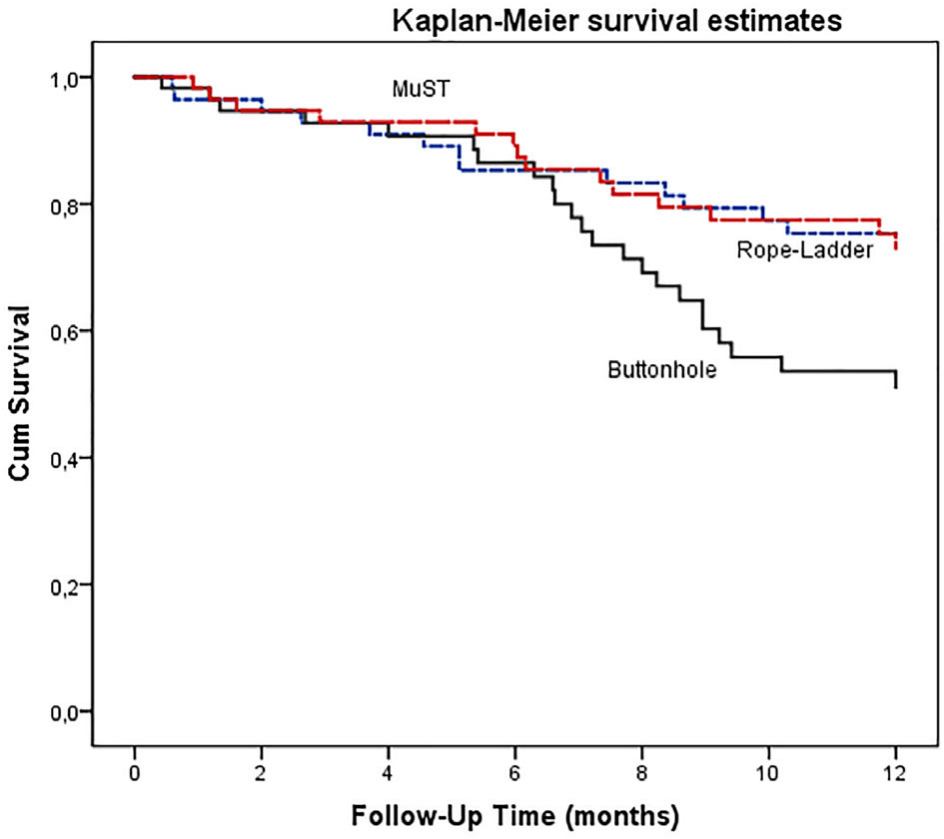
AVF survival curve comparing the three groups of cannulation technique, MuST versus BHC and versus RLC. (log‐rank test, *p* = 0.033). The Kaplan–Meier analysis showed that AVF survival during the 1st 6 months in the three groups was overlapping. After the 6th month, the BHC group survival curve decreased exponentially [Color figure can be viewed at wileyonlinelibrary.com]

The survival curve estimates are shown in Figure 4 (log rank test *p* = 0.033) and showed a significant difference in AVF survival between BHC and the other two groups. The three CT survival curves were overlapping in the first 6 months and they were exponential only after that period for BHC. The survival curves between MuST and RLC are equivalent. From the data daily reported by nurses, 4 (7.1%) patients abandoned RLC and 5 (8.8%) BHC, due to difficulties associated with the execution of the CT and the patient's refusal related to pain.

In Cox proportional‐hazards model, it was noted that BHC was a significant risk predictor (*p* = 0.03) in AVF survival with 2.1 times more events than the other two CT (HR = 2.13; 95% CI = 1.07, 4.21).

### Secondary outcomes

Inflammatory signs were only observed at the cannulation site in one patient who was using BHC. As reference factors for vascular surgery, one patient was reported with an exuberant aneurysm development associated with RLC. Aneurysm development was also reported in another two patients: one with RLC and one with MuST.

## DISCUSSION

This study is the first to exclusively include patients with new AVF, reducing bias in the selection of previously used fistulas. At the beginning of the study, training was provided to all nurses involved, which promoted a better compliance with the practices and records.

Results suggest that there is a significant difference in the primary patency of AVF cannulated with BHC compared with MuST and RLC. The survival curve was exponentially shorter in the BHC group after the 6th month of follow‐up. The main cause of referral for angiography in this group was the confirmed Qa drop in AVF output. Currently, there is no explanation for these findings, but they may be related to the presence of stenosis associated with BHC.^18^ The fact that multiple cannulators were used can cause multiple tracks and may lead to endothelial hyperplasia and possible stenosis.^19^

BHC was abandoned by five patients due to difficulties in forming or maintaining the track, limitations that have also been reported in previous studies.^9^, ^10^ It is important to mention this because BHC is the most used method of cannulation by home HD and self‐cannulation' patients. Effectively, this CT requires strict compliance with a set of rules that are not easy to implement. In addition, the use of blunt needles is also associated with insertion difficulties or missed cannulations^6^ and pain.^10^ So, in regarding to MuST, this CT also differs from BHC because it is not necessary the same angle of cannulation and thus the use of blunt needles and the same cannulator is unnecessary.

Four patients abandoned RLC due to pain during cannulation. As already mentioned, the participants included in this study did not apply local anaesthetic cream, and this CT is difficult to implement due to this factor.

Results also suggest that the MuST cannulation method is different from RLC and offered advantages to the nurses for its simplicity in execution.

No patient abandoned MuST CT related to pain or other factors. Associated with these advantages is that the use of local anaesthetic cream, blunt needles, and the same cannualtor can be dispensed with.

Several studies have reported a significant increase in infection risk associated with BHC cannulation^7^, ^8^, ^9^ when compared to RLC. However, only one patient in the BHC group exhibited inflammatory signs at the cannulation sites, 11.70 months after the study start. The use of topical antibiotic as a prophylaxis has been used with good results^9^; however, it was not used in this study. The excellent results suggest that are a relation with the implemented rules: hands and AVF arm washing along with patient training. In addition, the availability of all the disposables and equipment described above and compliance with the protocol led to a decrease in infection risk associated with cannulation. Local inflammatory signs or episodes of bacteraemia most likely occur due to the lack of disinfection measures in cannulation sites, or lack of conditions in facilities, particularly in studies with long follow‐up.^20^

Prior research suggested that BHC offered significant advantages to participants, such as reduction in existing aneurysm size^10^, ^21^ or less development of new aneurysm.^6^, ^10^

Conversely, a residual number of aneurysms in patients using the RLC was observed when compared to previous studies; thus, this study had no statistical power to detect differences in the incidence of aneurysm between groups. A possible explanation for the lower number of new aneurysms in the MuST group might be that this CT causes less vessel damage, due to consistently using the same site once a week. The study showed that on average, six patients punctured by MuST (instead BHC) can avoid that one additional patient with AVF, to have a primary outcome during a year of follow‐up.

Our study involved multiple units and hundreds of nurses, and only participants with new AVF were recruited. The three groups were homogeneous in the baseline variables described above. The follow‐up time or the number of recruited participants was a limitation that did not allow us to find significant differences in secondary outcomes between groups.

In conclusion, these results suggest that the use of MuST cannulation in HD patients was efficacious and safe, minimizing complications, and maintaining the longevity of AVF. The results of this study suggest that MuST might be an ideal CT for self‐cannulation and can be adapted for patients undergoing dialysis frequency more than three times a week. RLC using a diagram also proved to be a safe CT.

## CONFLICT OF INTEREST

Ricardo Peralta, João Fazendeiro Matos, Bruno Pinto, Pedro Gonçalves, Rui Sousa, Carla Felix, and Pedro Ponce are employees of Fresenius Medical Care and may hold shares in the company.
